# Genomic Landscape and Antimicrobial Resistance of *Listeria monocytogenes* in Retail Chicken in Qingdao, China

**DOI:** 10.3390/foods14183260

**Published:** 2025-09-19

**Authors:** Wei Wang, Yao Zhong, Juntao Jia, Lidan Ma, Yan Lu, Qiushui Wang, Lijuan Gao, Jijuan Cao, Yinping Dong, Qiuyue Zheng, Jing Xiao

**Affiliations:** 1 NHC Key Laboratory of Food Safety Risk Assessment, Division IV of Food Safety Standard, China National Center for Food Safety Risk Assessment, Beijing 100022, China; wangweiwsw@cfsa.net.cn (W.W.); xiaojing@cfsa.net.cn (J.X.); 2 College of Life Science, Dalian Minzu University, Dalian 116600, China; 15769345659@163.com (Y.Z.); caojijuan@dlnu.edu.cn (J.C.); 3 Technology Center of Qingdao Customs, Qingdao 266002, China; jiajt@163.com; 4 Technical Center of Gongbei Customs, Zhuhai 519015, China; 2212097@163.com; 5 Green Food Science Research Institute (National Research Center of Dairy Engineering and Technology), Northeast Agricultural University, Harbin 150086, China; judelily@126.com; 6 Institute of Analysis and Testing, Beijing Academy of Science and Technology (Beijing Center for Physical and Chemical Analysis), Beijing 100022, China; wangqius9182901@hotmail.com (Q.W.); aglj889@163.com (L.G.)

**Keywords:** whole genome sequencing, *Listeria monocytogenes*, genotyping, virulence gene, resistance gene

## Abstract

*Listeria monocytogenes* (*L. monocytogenes*) is an important foodborne pathogen that poses great risks to food safety and public health, and knowledge about its presence and diversity in potential sources is crucial for effectively tracking and controlling it in the food chain. In this study, we investigated the prevalence, antimicrobial susceptibility, and genomic characteristics of *Listeria monocytogenes* (*L. monocytogenes*) collected from retail chicken meat samples in Qingdao, China, in 2022. A total of 38 (10.6%, 38/360) *L. monocytogenes* isolates were recovered from 360 retail chickens. All 38 isolates were classified into two lineages (I and II), three serogroups (IIa, IIb, IIc), eight sequence types (STs), eight clonal complexes (CCs), eight Sublineages (SLs) and nine cgMLSTs (CTs). ST121 and ST9 were the most prevalent STs in this study. The ST121 strains from China had heterogeneity with those from other countries, while the Chinese ST9 strains had homogeneity with those from other countries. One resistance cassette *tet(M)-entS-msr(D)* was identified in eight L2-SL121-ST121-CT13265 isolates, the genetic structure of which was identical to that of three reference genomes. All isolates carried the *L. monocytogenes* pathogenic island (LIPI)-1, with only one carrying LIPI-3 and three carrying LIPI-4. In addition, 11 isolates subtyped as L2-SL121-ST121-CT13265 were found to have a premature stop codon (PMSC) in the *inlA* gene in this study. Our data revealed the antimicrobial susceptibility, genomic characteristics and evolutionary relationships of *L. monocytogenes* in retail chicken in Qingdao, China. The characterization of genotypes, virulence, stress and antimicrobial markers of strains circulating in retail chicken in Qingdao, as described in this study, provides the opportunity to improve risk assessments of *L. monocytogenes* exposure.

## 1. Introduction

*Listeria monocytogenes* (*L. monocytogenes*) is widely distributed in the environment and is commonly transmitted to humans through the consumption of contaminated foods, including meat, poultry, dairy, fish, and vegetable products [1]. It is the causative agent of listeriosis, an intracellular disease that predominantly affects the elderly, immunosuppressed people, and pregnant women along with their unborn or newborn babies. Although the incidence of the disease is low compared to other food-borne pathogens [2], the disease outcome is often more serious, making it a priority pathogen in many countries. In the USA, the Center for Disease Control (CDC) has estimated that 1600 people are subjected to listeriosis each year, with approximately 260 people dying from the disease [3]. In 2021, the European Union reported 2183 confirmed invasive human listeriosis cases, resulting in 196 fatalities. This figure reflects a stabilization in case numbers from 2017 to 2021, following a previous prolonged period of increase [4]. Meanwhile, in China, 253 invasive listeriosis cases were documented across 19 provinces between 2011 and 2016, with an overall case–fatality rate of 25.7% [5]. Owing to its environmental ubiquity and high mortality, *L. monocytogenes* remains a significant public health and food safety concern [6]. Unlike other pathogenic bacteria such as *Salmonella* and *Staphylococcus aureus*, which are commonly found in food, *L. monocytogenes* is more tolerant to acids and salts, can survive at low temperatures, and generates biofilms, a property that allows the bacterium to survive in more complex environments, and has a mechanism for developing resistance to bactericides [7].

At present, *L. monocytogenes* clusters into at least four lineages (lineage I to lineage IV), divided into thirteen serotypes [8]. Lineage I isolates are predominantly associated with human listeriosis, while lineage II isolates are over-represented in food products and food-associated environments, but also implicated in a number of major listeriosis outbreaks [9,10]. Lineages III and IV isolates are rare and commonly isolated from animal sources [11]. Moreover, multi-locus sequence typing (MLST) is widely employed as a standard genotyping method for comparing distinct clonal groups of *L. monocytogenes* based on nucleotide sequence variations in housekeeping genes. Predominant isolates from food and human sources exhibit distinct distributions. Specifically, clonal complexes CC1, CC2, CC4, and CC6 are associated with infections and are frequently implicated in both sporadic cases and outbreaks of listeriosis. In contrast, CC9 and CC121 are predominantly linked to food sources and often infect immunocompromised individuals. [12]. At the same time, the pathogenicity of *L. monocytogenes* is also determined by virulence factors, including internalin A (*inlA*), endotoxin encoded by internalin B (*inlB*), *L. monocytogenes* pathogenicity island 1 (LIPI-1), *Listeria* pathogenicity island 3 (LIPI-3) and *Listeria* pathogenicity island 4 (LIPI-4) clustered along chromosomes [13], invasion-related proteins encoded by hemolysin A (*hlyA*) and invasion-associated proteins encoded by cell wall hydrolase (*iap*) [14]. In the epidemiological investigation of this bacterium, whole genome sequencing (WGS) can help subtype different strains to identify bacterial species, strains, and genotypes, as well as understand strain evolution and phylogenies. Meanwhile, WGS can also predict the potential antimicrobial resistance and pathogenicity of strains by analyzing virulence, antimicrobial resistance (AMR) genes and point mutations in the genomes [15]. Currently, the WGS technique has been used in both cultured organisms and directly in clinical specimens. *L. monocytogenes* isolates characterized in frozen foods by WGS showed that most of the mononuclear *L. monocytogenes* isolates were identified as ST7 [16]. Tirloni et al. demonstrated the virulence and pathogenicity of *L. monocytogenes* isolates by WGS [17].

The extensive usage of antimicrobial agents has increased AMR in bacterial pathogens and the spread of resistant pathogens through the food chain, leading to an increased global public health threat in terms of morbidity, mortality, and cost of treatments [18]. AMR *L. monocytogenes* has been frequently detected since a multi-drug-resistant (MDR) strain was found in France in 1988 [19]. In China, it is reported that the average resistance rate of 1687 *L. monocytogenes* against 15 antimicrobial agents is 6.82%, and that tetracycline resistance is the most prevalent, accounting for 5.69% [20]. The emergence and dissemination of AMR in *L. monocytogenes* has been one of the serious problems for the treatment of infectious diseases. Moreover, the prevalence of *L. monocytogenes* in raw meat and its derived products is higher than that in any other foods [21]. It was reported that *L. monocytogenes* was more likely to be isolated from chicken meat than pork or beef [2]. In this study, *L. monocytogenes* was isolated from retail chicken meat samples in Qingdao, China, in 2022. Isolates were obtained for antimicrobial susceptibility testing and WGS was used to extend the characterization of the AMR and virulence genotypes and to improve food safety controls.

## 2. Materials and Methods

### 2.1. Bacterial Isolation

In total, 360 retailed chilled chicken meat samples were collected from local markets in Qingdao, China, in 2022. All samples were screened for *L. monocytogenes* using a qualitative method according to the National Food Safety Standard of China—Food microbiological examination, *L. monocytogenes* (GB 4789.30-2016) [22]. Subsequently, the presumptive *L. monocytogenes* was confirmed by API *Listeria* test identification strips (bioMérieux, Marcy l’Etoile, France) and a PCR assay targeting the *hlyA* gene [23]. All of the confirmed *L. monocytogenes* isolates were used for this study.

### 2.2. Antibiotic Susceptibility Testing

The susceptibility of all *L. monocytogenes* isolates in this study was determined using a broth micro-dilution method described by the Clinical and Laboratory Standards Institute (CLSI, Third Edition: M45) [24]. Nine antimicrobial agents were tested, including ampicillin (AMP), clindamycin (CLI), ciprofloxacin (CIP), erythromycin (ERY), penicillin (PEN), meropenem (MEM), trimethoprim/sulfamethoxazole (SXT), tetracycline (TET) and vancomycin (VAN). All antimicrobials were purchased from Sigma-Aldrich, Germany. The *Streptococcus pneumoniae* ATCC49619 and *Staphylococcus aureus* ATCC29213 strains were used as the control for the antibiotic susceptibility testing.

### 2.3. Preparation of Genome DNA

Each studied *L. monocytogenes* isolate was enriched overnight in brain heart infusion (BHI) broth (HopeBio) at 37 °C, and the genomic DNA (gDNA) of each isolate was purified using an Omega EZNA Bacterial DNA kit (Omega Bio-Tek, Norcross, GA, USA) according to the manufacturer’s procedures. The harvested DNA was qualified by agarose gel electrophoresis and quantified by a Qubit 2.0 fluorometer (Thermo Fisher Scientific, Waltham, MA, USA).

### 2.4. Whole-Genome Sequencing of L. monocytogenes Isolates

The DNA sample of each *L. monocytogenes* isolate was fragmented by sonication to a size of 350 bp; it was then end polished, tailed and ligated with the full-length adaptor for sequencing with further PCR amplification. Finally, PCR products were purified (AMPure XP system), and the size distribution of DNA libraries was analyzed by an Agilent 2100 Bioanalyzer and quantified using RT-PCR. The resultant DNA preparations were sequenced using an Illumina HiSeq 1000 at Beijing Novogene Bioinformatics Technology. Trimmingomatic (Beijing, China) [25], FastQC (https://www.bioinformatics.babraham.ac.uk/projects/fastqc) (accessed on 14 August 2023), SPAdes v3.14 [26], and Prokka v1.14.5 [27] were used for read quality control, assembly, and annotation.

### 2.5. In Silico Subtyping and Phylogenetic Analysis

To elucidate the genetic relationships and population structure of the isolates, multiple molecular typing schemes were computationally assessed via the BIGSdb-Lm platform (https://bigsdb.pasteur.fr/listeria) (accessed on 20 November 2023), including the PCR-serogroup, serotype, phylogenetic lineage, multilocus sequence typing (MLST), clonal complex (CC), and core genome MLST (cgMLST) type (CT). Sublineage (SL) and CT assignments were inferred directly within the database using whole-genome cgMLST profiles, with the allelic difference thresholds (≤150 alleles for SL and ≤7 alleles for CT) established as defined in reference [28]. For broader phylogenetic reconstruction, all annotated genomes were subjected to pan-genome analysis using Roary v3.13 [29] to identify and align core genes. A maximum-likelihood phylogeny was inferred from the core genome alignment with IQ-TREE v2.0.3 [30] under the general time reversible (GTR) model, with the rate heterogeneity accounted for by a FreeRate model (1F1R10). The resulting tree was annotated and visualized using iTOL [31]. To resolve the population structure within individual sequence types (STs), a dedicated core genome phylogenetic analysis was performed for each ST incorporating publicly available reference genomes from the BV-BRC database [32] (Dataset S1). For each ST-specific dataset, core genes were extracted and aligned using Roary [29]. Phylogenetic trees were then reconstructed under the maximum-likelihood framework in IQ-TREE v2.0.3 [30] based on concatenated core gene alignments, with final topological visualization conducted in iTOL [31].

### 2.6. Genome Annotation

The stress resistance, virulence factors and antimicrobial resistance genes were identified by comparing with the BIGSdb-Lm database, with a minimum of 90% coverage and 90% identity [33]. The comparation of AMR genes with reference genomes was performed by Easyfig v2.2.2 [34]. The truncated *inlA* (premature stop codon, PMSC) among the studied *L. monocytogenes* was identified by blast against the *inlA* protein of the EGD-e isolate (NC_003210.1) [35].

## 3. Results

### 3.1. Prevalence and Antimicrobial Susceptibility of L. monocytogenes in Chicken Meats

**Figure 1:**
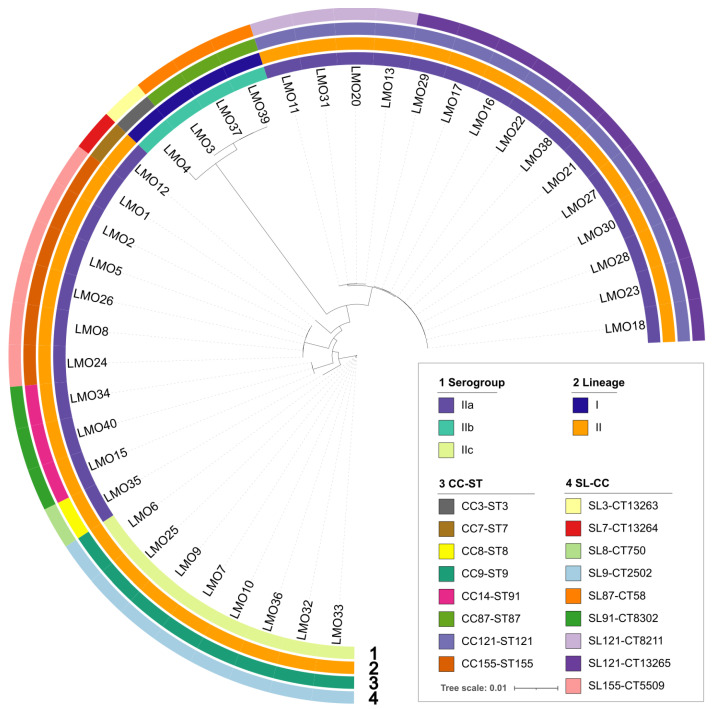
Phylogenetic tree and genomic characterization of 38 *L. monocytogenes* isolated from retailed chilled chicken meat samples from local markets in Qingdao, China, in 2022. Information about serogroup, lineage, clonal complex and sequence type (CC-ST), SLs and cgMLST (SL-CC) are provided on the right and marked with a different color.

Of the 360 retailed chilled chicken meat samples collected from local markets in Qingdao, China, in 2022, 38 samples were positive for *L. monocytogenes*, yielding a detection rate of 10.6% (38/360). The susceptibility patterns of all 38 *L. monocytogenes* isolates were determined against nine antimicrobials using broth micro-dilution. All isolates were susceptible to most antibiotics tested; however, eight (21.1%, 8/38) exhibited resistance to both erythromycin and tetracycline (Figure 1, Table 1 and Supplementary Table S1).

### 3.2. L. monocytogenes Population Structure

The 38 *L. monocytogenes* isolates were grouped by lineage I (4/38, 10.5%) and lineage II (34/38, 89.5%), and by serogroups IIa (26/38, 68.4%), IIb (4/38, 10.5%) and IIc (8/38, 21.1%) (Figure 1 and Supplementary Table S1). Based on 7-loci MLST, isolates were distributed among eight different sequence types (STs) and eight clonal complexes (CCs) (Figure 1, Table 2 and Supplementary Table S1). Among these, ST121 (serogroup IIa, *n* = 15), ST9 (IIc, *n* = 8) and ST155 (IIa, *n* = 6) were the top three detected STs, followed by ST87 (IIb, *n* = 3) and ST91 (IIa, *n* = 3). In addition, one of each isolate was identified as ST3, ST7, and ST8.

The core genome phylogeny delineated nine unique cgMLSTs, categorizing the isolates across eight different SLs (Figure 1 and Supplementary Table S1). The SLs and cgMLSTs were consistent with the MLST results, with the exception that the 15 ST121 isolates were sub-typed as 4 CT8211 and 11 CT13265 (Figure 1 and Supplementary Table S1). Three *L. monocytogenes* clones of L2-SL121-ST121-CT13265 (*n* = 11), L2-SL9-ST9-CT2502 (*n* = 8), and L2-SL155-ST155-CT5509 (*n* = 6) were the dominant clones identified in this study, followed by L1-SL87-ST87-CT58 (*n* = 3), L2-SL91-ST91-CT8302 (*n* = 3), and L2-SL121-ST121-CT8211 (*n* = 4).

### 3.3. The Distribution of the Prevalent Sequence Types of L. monocytogenes Isolates from Food in China in the Context of Global Isolates

**Figure 2:**
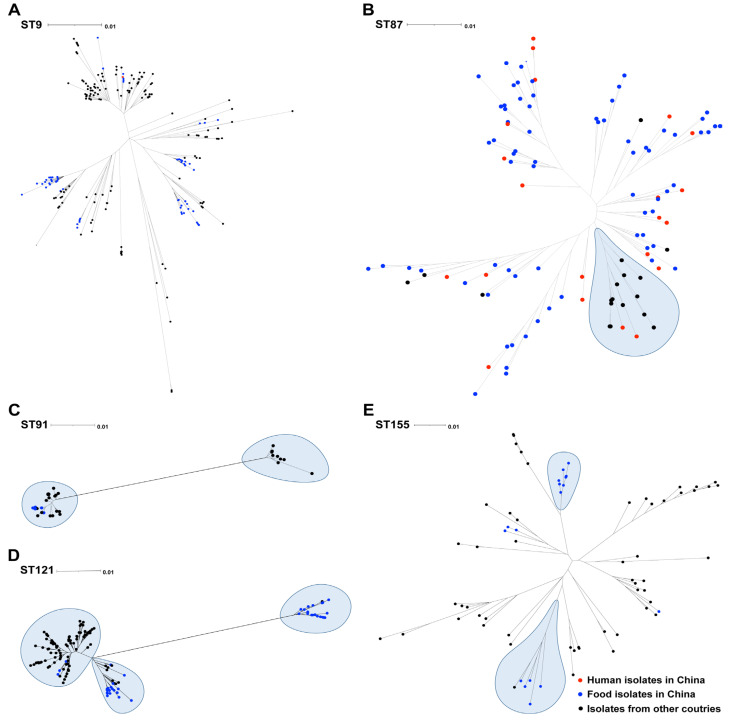
Phylogenetic tree of the prevalent five STs of *L. monocytogenes* in China in the context of global isolates. (**A**) Phylogenetic tree of the prevalent ST9 of *L. monocytogenes* in China in the context of global isolates; (**B**) Phylogenetic tree of the prevalent ST87 of *L. monocytogenes* in China in the context of global isolates; (**C**) Phylogenetic tree of the prevalent ST91 of *L. monocytogenes* in China in the context of global isolates; (**D**) Phylogenetic tree of the prevalent ST121 of *L. monocytogenes* in China in the context of global isolates; (**E**) Phylogenetic tree of the prevalent ST155 of *L. monocytogenes* in China in the context of global isolates. Human isolates in China are represented by a red ball, food isolates in China are represented by a blue ball, and isolates from other countries are represented by a black ball.

To contextualize the dominant STs identified here, namely ST9, ST87, ST91, ST121, and ST155, within broader geographic populations, we compared them against 572 publicly available *L. monocytogenes* genomes from China and other countries (Figure 2 and Dataset S1). We constructed individual phylogenetic trees for each ST from aligned core genome sequences (Figure 2). For ST91, the recruited Chinese *L. monocytogenes* isolates clustered together and separated from the international isolates. However, for other STs (ST9, ST87, ST121, and ST155), the *L. monocytogenes* isolates from China were found to be mixed with the reference isolates from other regions, while no spatial or source specificity was found.

### 3.4. Antimicrobial Resistance Genes in the Studied L. monocytogenes Isolates

**Figure 3:**
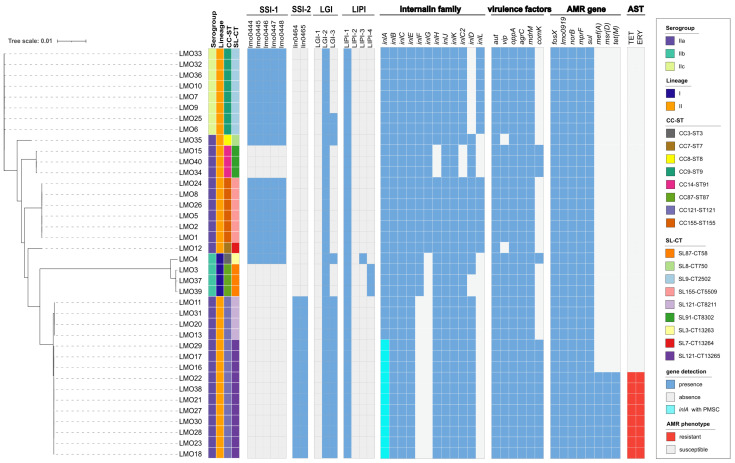
Phylogenetic tree and genomic characterization of 38 *L. monocytogenes* isolated from retailed chilled chicken meat samples from local markets in Qingdao, China, in 2022. The presence of different genes or resistant phenotypes is marked with a red box, while the truncated *inlA* gene (PMSC) is represented in bright blue.

In total, eight AMR genes were identified in this study (Figure 3 and Supplementary Table S1). We detected five intrinsic antimicrobial resistance genes, namely *fosX* (resistance to fosfomycin), *lmo0919* (lincosamides), *norB* (quinolones), *mprF* (cationic antimicrobial peptides), and *sul* (sulfonamides), in all isolates, which mediate resistance to fosfomycin, lincosamides, quinolones, cationic antimicrobial peptides, and sulfonamides, respectively. One resistance cassette containing three acquired AMR genes of *tet(M)-entS-msr(D)* was identified in eight isolates (all were sub-typed as L2-SL121-ST121-CT13265), all of which showed resistance to both erythromycin and tetracycline.

**Figure 4:**
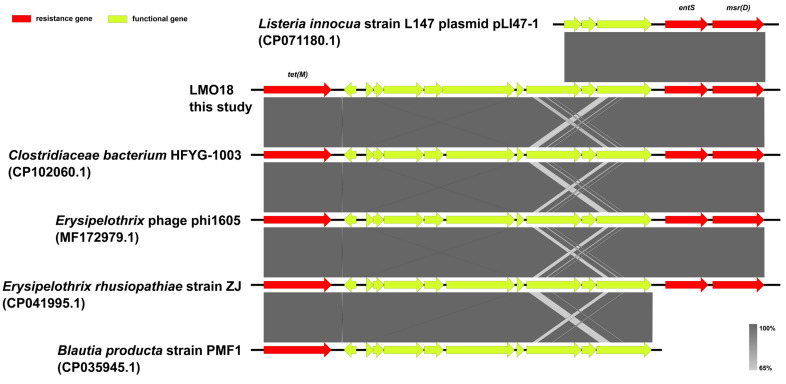
Linear sequence comparison of the *tet(M)-entS-msr(D)* resistance cassette with the reference genomes. Gray shading represents regions of homology. CDSs are shown as arrows. Antimicrobial resistance genes are highlighted in red. Other functional genes are highlighted in yellow-green.

Moreover, the contigs carrying the *tet(M)-entS-msr(D)* cassette were genetically identical across all eight resistant isolates; a representative contig from isolate LMO18 was therefore selected for subsequent analysis. Linear sequence comparison was performed using the resistance contig of LMO18 with five public genomes as the closest common hits of the online BLAST v2.12.0 results against the NCBI nt/nr database (Figure 4). This *tet(M)-entS-msr(D)* cassette was almost identical to the cassettes among the reference genomes of Clostridiacae bacterium HFYG-1003 (CP102060.1), Erysipelothrix rhusiopathiae strain ZJ (CP041995.1), and Erysipelothrix phage phi1605 (MF172979.1).

### 3.5. Assessment of Virulence Factor Profiles and Stress Islands

In this study, *L. monocytogenes* isolates were screened for the presence of key stress-related genetic elements, such as stress survival islets 1 and 2 (SSI-1, SSI-2), three *Listeria* genomic islands (LGI-1, LGI-2, LGI-3), and four virulence islands (LIPI-1, LIPI-2, LIPI-3, LIPI-4) (Figure 3). SSI-1 was detected in 45% of the 38 isolates (17/38), with 16 in lineage II, and only 1 in lineage I. SSI-2 was detected in 39% of the 38 isolates (15/38), all of which belonged to serogroup IIa, lineage II, CC121-ST121, and SL121 (11 CT13265 and 4 CT8211). LIPI-1 was highly conserved among all isolates, carrying LGI-2, and 19 isolates carried LGI-3. LIPI-3 was identified in only one isolate belonging to a subset of lineage I, while LIPI-4 was identified in three CC87-ST87 isolates.

**Figure 5:**
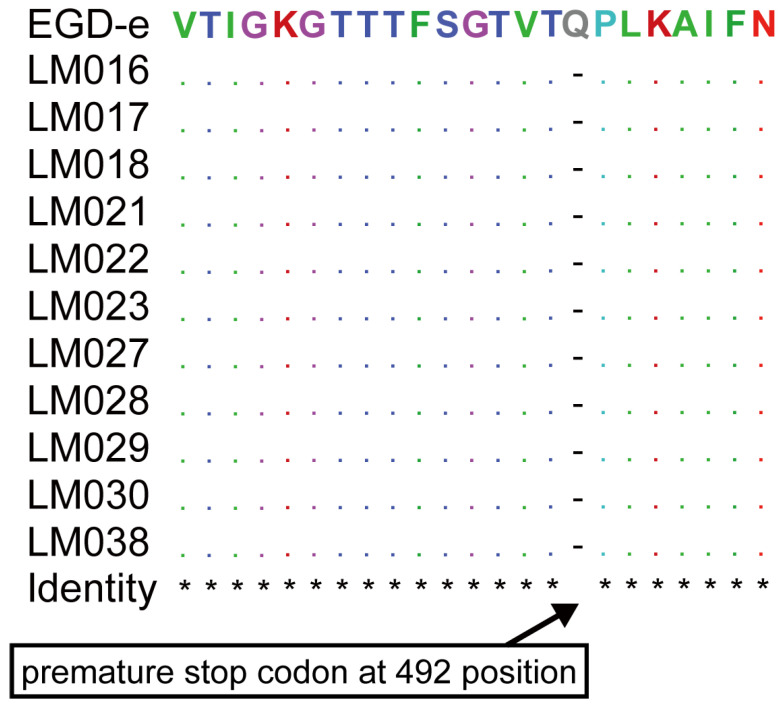
Strain sequences of truncated *inlA* alleles with PMSC due to point mutation. Symbol * means the amino acid sequences are consistent with that of the *L. monocytogenes* EGD-e strain.

The intact *inlA* gene encoding full-length internalin A (*inlA*) was detected in 38 *L. monocytogenes* isolates, of which 11 lineage II isolates (L2-SL121-ST121-CT13265) were truncated due to premature stop codon (PMSC) mutations, and the position of PMSC in the isolate was 492 (Figure 5). Of the internalin family genes, the *inlB*, *inlC*, *inlE*, *inlJ*, and *inlK* genes were found in all strains studied, while the other genes were absent in several isolates. For instance, the *inlD* gene was absent in all CC9-ST9 isolates, the *inlH*, *inlC2* and *inlL* genes were absent in all the CC14-ST91 isolates, and the *inlF* and *inlL* genes were absent in all the CC121-ST121 and lineage II isolates (Figure 3 and Supplementary Table S1).

In addition, the virulence gene aut was found in all the strains studied, and the *vip* gene was not present in ST8 and ST7 isolates. In addition, the *oppA*, *agrC*, and *mdrM* genes were detected in all isolates, and the *comK* gene was detected in ST3, ST91, and ST121 isolates (Figure 3 and Supplementary Table S1).

## 4. Discussion

In this study, the WGS analysis revealed that all of the 38 *L. monocytogenes* belonged to two lineages (I and II), 35 strains belonged to lineage II, 4 strains belonged to lineage I, and no strains belonged to lineages III and IV. Accordingly, the vast majority of *L. monocytogenes* belong to lineage I and lineage II, and different genetic lineages have different strain virulence potential. Of these, the most widespread are lineage I, which includes serogroups IIb and IVb, which are often associated with human cases of listeriosis; lineage II, including serotypes IIa and IIc, which are more common in food and food processing environments, is less pathogenic than lineage I strains [36,37]. The sample was procured from retailed chilled chicken meat from local markets in Qingdao, China, where the breeding and consumption of chicken are relatively high. According to unpublished data, only in the first quarter of 2025 alone did the number of broiler chickens produced in Qingdao reach nearly 20 million. However, few data were available on the contamination and genomic characteristics of *L. monocytogenes* among chicken meat in Qingdao, leading to obstacles in food safety risk prevention and control. Our study fills this gap to some extent, providing data for the epidemiological study and risk assessment of foodborne *L. monocytogenes* in Qingdao.

*L. monocytogenes* microbial monitoring now relies on WGS and universally applicable genome-wide strain genotyping approaches, such as cgMLST. This WGS-based cgMLST method significantly enhances the detection of clusters of microbiologically relevant listeriosis cases [27], while also supplying the academic community with extensive genomic data from sequenced *L. monocytogenes* isolates [38]. cgMLST is based on 1748 core genes for typing, which separates strains of different lineages or CC types, and uses more abundant genetic information and a higher resolution than MLST. The 38 strains in this study belonged to nine cgMLSTs, with the dominant types being CT2502, CT5509, and CT13265, while CT8211 included only four strains; CT8302, CT58, CT750, CT13264, and CT13263 contained only three and one strain, respectively. The lineage, ST, and CC type of strains with the same cgMLST were also identical. The diversity and distribution observed in this study were consistent with those previously described in a globally representative dataset [9,10].

A total of 572 global *L. monocytogenes* genomes from 23 countries were applied for comparative analysis. There have been several reports on the isolation of *L. monocytogenes* from chicken in these countries, as well as the foodborne diseases caused by this bacterium. For instance, in the USA, chicken salad was reported to be the source of *L. monocytogenes* infections [39], while in the Netherlands and Italy, 19.4% and 15% of human listeriosis cases could be attributed to chicken, respectively [40,41]. Although few outbreaks of listeriosis have been reported in China, *L. monocytogenes* was frequently detected in chicken [42]. Therefore, the prevalence of *L. monocytogenes* in chicken should receive more attention.

As well as affording high-resolution typing and phylogenetic context, WGS provides immediate access to a wealth of additional data. AMR in *Listeria* spp. has been studied in various food, environmental and clinical settings [5,18]. In this study, 21.1% (8/38) of the 38 *L. monocytogenes* isolates showed resistance to both erythromycin and tetracycline, which is much higher than those previously reported [37]. The WGS analysis revealed a *tet(M)-entS-msr(D)* resistance cassette identified from eight L2-SL121-ST121-CT13265 isolates, which also have the same virulence profiles. The macrolide resistance genes *mefA* and *msrD*, as well as the tetracycline resistance gene *tetM*, have been previously reported in *L. monocytogenes* [43]. However, to the best of our knowledge, our study was the first to report this *tet(M)-entS-msr(D)* resistance cassette. Notably, this cassette was almost identical to those among the reference genomes from genera other than *Listeria* spp. Because *L. monocytogenes* has generally been shown to be more susceptible to antimicrobial agents than other species [44], it can be inferred that this resistance cassette might have been acquired by *L. monocytogenes* from other species during evolution. It is therefore imperative to maintain vigilance against this emerging form of resistance.

The virulence gene of *L. monocytogenes* was precisely regulated at each stage of infection. The analysis of virulence factors carried by *L. monocytogenes* strains showed that the virulence genes carried by different CC strains were quite different, while the distribution of virulence genes of the same CC strains was similar. In this study, LIPI-1 was stable in all strains, with the CC3 isolate carrying the intact LIPI-3 gene and CC87 isolate carrying the intact LIPI-4 gene. It has been reported that the clonal groups that cause listeriosis in China are mainly CC87, CC8 and CC9, among which CC87 carries the virulence islands LIPI-1 and LIPI-3, which is one of the highly pathogenic clonal groups prevalent in clinical isolates [45]. In this study, all isolates had *aut* genes, but *vip* genes were not present in CC8 and CC7 isolates. These virulence genes found in *L. monocytogenes* play an important role in the infection process of the host, and surface proteins such as *vip* are the main adhesion and invasion virulence factors, mediating bacterial invasion into cells [46].

The *inlA* gene, one of the primary virulence factors belonging to the internalin protein family, enables *L. monocytogenes* to cross the intestinal barrier and invade epithelial cells [47]. However, PMSC mutations in the *inlA* gene may lead to truncated *inlA* that cannot be anchored to the bacterial cell wall, reducing the bacterium’s ability to invade human intestinal epithelial cells in vitro [48]. Accordingly, most clinical isolates have intact *inlA*, while *inlA* with PMSCs is more prevalent in environmental and food-related isolates [49]. Notably, 11 isolates, all identified as L2-SL121-ST121-CT13265, were found to carry truncated *inlA* due to a PMSC mutation at position 492. This finding is consistent with previous reports [50]. The results reveal the distribution of virulence potential among *L. monocytogenes* circulating in the Qingdao area and could help improve risk assessment approaches.

In addition, the distribution of stress survival islands (SSI-1 and SSI-2) further reflects the differential adaptive potential of *L. monocytogenes* strains to environmental stressors. SSI-1 was detected in 45% of the strains, predominantly within lineage II, while SSI-2 was exclusively identified in strains belonging to serogroup IIa, lineage II, CC121-ST121, and sublineage SL121. The presence of these genetic elements is likely to enhance bacterial resilience under food processing and storage conditions, potentially increasing the risk of transmission through the food chain.

From a food safety and public health perspective, this study underscores the necessity of establishing a genomics-based surveillance system to continuously monitor *L. monocytogenes*, with particular emphasis on the co-dissemination of antimicrobial resistance and virulence genes. Future studies should aim to expand sample sizes and diversify sources, integrating phenotypic assays and epidemiological investigations to elucidate the evolutionary dynamics and risk factors associated with *L. monocytogenes* at the food–human interface. Such insights will be critical for developing targeted and effective prevention and control strategies.

## 5. Conclusions

In this study, WGS was used to analyze the characteristics of *L. monocytogenes* isolates such as AMR genes and virulence factors to better understand their pathogenicity. The structural characteristics of the *L. monocytogenes* genomes provide basic data for long-term monitoring and rapid traceability, and also provide a reference for the applicable scenarios of different monitoring methods. At the same time, attention should be paid to the potential resistance and pathogenic risk of *L. monocytogenes*. WGS allows antimicrobial resistance and virulence monitoring to be performed at no additional cost if WGS is part of routine microbial surveillance and, therefore allows this potential threat to be monitored going forward. The characterization of the genotypes, virulence, stress and antibiotic markers of strains circulating in retail chicken in Qingdao, as described in this study, provides the opportunity for improved risk assessments for *L. monocytogenes* exposure. Notably, this study also highlighted that the presence of SSI-2 and resistance, as well as PMSC in *inlA,* appears to be characteristic of the isolates of lineage II. For instance, the dominance of specific clones, such as L2-SL121-ST121-CT13265, could suggest the presence of persistent strains within processing facilities in the Qingdao area. Therefore, in the future, it is essential to focus on these high-risk clones to develop targeted sanitation protocols, enhance monitoring strategies, and improve risk assessment measures aimed at controlling *L. monocytogenes* contamination in the food supply chain.

## Figures and Tables

**Table 1:** Antimicrobial resistance rate of 38 *L. monocytogenes* isolates from retailed chilled chicken meat samples.

Antimicrobial Agent	Resistant (*n* = 38)	
	*n*	%
erythromycin	8	21.1%
tetracycline	8	21.1%
trimethoprim/sulfamethoxazole	0	0.0%
ciprofloxacin	0	0.0%
ampicillin	0	0.0%
penicillin	0	0.0%
vancomycin	0	0.0%
meropenem	0	0.0%
clindamycin	0	0.0%

**Table 2:** Genomic and virulence subtyping of 38 *L. monocytogenes* isolates from retailed chilled chicken meat samples.

Phylogenetic Lineage/Serogroups	CC-ST	SL-CT	SSI	LGI	LIPI	No. of Isolates
lineage I/IIb	CC87-ST87	SL87-CT58	ND ^a^	LGI-2	LIPI-1/LIPI-4	3
	CC3-ST3	SL3-CT13263	SSI-1	LGI-2/LGI-3	LIPI-1/LIPI-3	1
lineage II/IIa	CC121-ST121	SL121-CT13265	SSI-2	LGI-2/LGI-3	LIPI-1	11
	CC121-ST121	SL121-CT8211	SSI-2	LGI-2/LGI-3	LIPI-1	4
	CC14-ST91	SL91-CT8302	ND ^a^	LGI-2	LIPI-1	3
	CC155-ST155	SL155-CT5509	SSI-1	LGI-2	LIPI-1	6
	CC7-ST7	SL7-CT13264	SSI-1	LGI-2	LIPI-1	1
	CC8-ST8	SL8-CT1750	SSI-1	LGI-2/LGI-3	LIPI-1	1
lineage II/IIc	CC9-ST9	SL9-CT2502	SSI-1	LGI-2	LIPI-1	6
	CC9-ST9	SL9-CT2502	SSI-1	LGI-2/LGI-3	LIPI-1	2

^a^ ND means no detection.

## Data Availability

The sequences obtained in this study have been deposited in the NGDC Genome Sequence Archive (https://ngdc.cncb.ac.cn/gsa/) (accessed on 25 February 2025) under accession number CRA023295. All accession numbers of the publicly available genomes were available in Supplementary Table S1 and Dataset S1. All reference genomes used in this study were retrieved from NCBI, all of which were publicly available and unrestricted re-use.

## Supplementary Materials

The following supporting information can be downloaded at: https://www.mdpi.com/article/10.3390/foods14183260/s1, Supplementary File S1: Data set; Table S1: Summary of the isolation, AMR and genomic characteristics of *Listeria monocytogenes* collected from retailed chicken meat in Qingdao, China, in 2022.

## References

[1] Meloni D. (2015). Presence of *Listeria monocytogenes* in mediterranean-style dry fermented sausages. Foods.

[2] Hernandez-Milian A., Payeras-Cifre A. (2014). What is new in listeriosis?. Biomed Res. Int..

[3] CDC Listeria (Listeriosis)|Listeria. https://www.cdc.gov/Listeria/outbreaks/index.html.

[4] European Food Safety Authority, European Centre for Disease Prevention Control (2022). The European Union One Health 2021 Zoonoses Report. EFSA J..

[5] Li W., Bai L., Fu P., Han H., Liu J., Guo Y. (2018). The Epidemiology of *Listeria monocytogenes* in China. Foodborne Pathog. Dis..

[6] Beresford M.R., Andrew P.W., Shama G. (2001). *Listeria monocytogenes* adheres to many materials found in food-processing environments. J. Appl. Microbiol..

[7] Mazaheri T., Cervantes-Huamán B.R.H., Bermúdez-Capdevila M., Ripolles-Avila C., Rodríguez-Jerez J.J. (2021). *Listeria monocytogenes* biofilms in the food industry: Is the current hygiene program sufficient to combat the persistence of the pathogen?. Microorganisms.

[8] Ragon M., Wirth T., Hollandt F., Lavenir R., Lecuit M., Le Monnier A., Brisse S. (2008). A new perspective on *Listeria monocytogenes* evolution. PLoS Pathog..

[9] Cruz C.D., Pitman A.R., Harrow S.A., Fletcher G.C. (2014). *Listeria monocytogenes* associated with New Zealand seafood production and clinical cases: Unique sequence types, truncated InlA, and attenuated invasiveness. Appl. Environ. Microbiol..

[10] Manuel C.S., Van Stelten A., Wiedmann M., Nightingale M., Orsi R.H. (2015). Prevalence and distribution of *Listeria monocytogenes inlA* alleles prone to phase variation and *inlA* alleles with premature stop codon mutations among human, food, animal, and environmental isolates. Appl. Environ. Microbiol..

[11] Leclercq A., Chenal-Francisque V., Dieye H., Cantinelli T., Drali R., Brisse S., Lecuit M. (2011). Characterization of the novel *Listeria monocytogenes* PCR serogrouping profile IVb-v1. Int. J. Food Microbiol..

[12] Maury M.M., Bracq-Dieye H., Huang L., Vales G., Lavina M., Thouvenot P., Disson O., Leclercq A., Brisse S., Lecuit M. (2019). Author Correction: Hypervirulent *Listeria monocytogenes* clones’ adaptation to mammalian gut accounts for their association with dairy products. Nat. Commun..

[13] Matle I., Mbatha K.R., Madoroba E. (2020). A review of *Listeria monocytogenes* from meat and meat products: Epidemiology, virulence factors, antimicrobial resistance and diagnosis. Onderstepoort J. Vet. Res..

[14] Abdeen E.E., Mousa W.S., Harb O.H., Fath-Elbab G.A., Nooruzzaman M., Gaber A., Alsanie W.F., Abdeen A. (2021). Prevalence, antibiogram and genetic characterization of *Listeria monocytogenes* from food products in Egypt. Foods.

[15] Singh R., Kusalik A., Dillon J.R. (2022). Bioinformatics tools used for whole-genome sequencing analysis of *Neisseria gonorrhoeae*: A literature review. Brief. Funct. Genom..

[16] Truchado P., Gil M.I., Querido-Ferreira A.P., Capón C.L., Álvarez-Ordoñez A., Allende A. (2022). Frozen vegetable processing plants can harbour diverse *Listeria monocytogenes* populations: Identification of critical operations by WGS. Foods.

[17] Tirloni E., Bernardi C., Pomilio F., Torresi M., De Santis E.P.L., Scarano C., Stella S. (2020). Occurrence of *Listeria* spp. and *Listeria monocytogenes* isolated from PDO Taleggio production plants. Foods.

[18] Oliveira M., Antunes W., Mota S., Madureira-Carvalho Á., Dinis-Oliveira R.J., Dias da Silva D. (2024). An overview of the recent advances in antimicrobial resistance. Microorganisms.

[19] Poyart-Salmeron C., Carlier C., Trieu-Cuot P., Courtieu A.L., Courvalin P. (1990). Transferable plasmid-mediated antibiotic resistance in *Listeria monocytogenes*. Lancet.

[20] Swaminathan B., Gerner-Smidt P. (2007). The epidemiology of human listeriosis. Microbes Infect..

[21] Ochiai Y., Yamada F., Batmunkh O., Mochizuki M., Takano T., Hondo R., Ueda F. (2010). Prevalence of *Listeria monocytogenes* in retailed meat in the Tokyo metropolitan area. J. Food Prot..

[22] (2016). National Food Safety Standard of China-Food Microbiological Examination, *Listeria monocytogenes*.

[23] Liu P., Mizue H., Fujihara K., Kobayashi H., Kamikado H., Tanaka T., Honjoh K., Miyamoto T. (2012). A new rapid real-time PCR method for detection of *Listeria monocytogenes* targeting the *hlyA* gene. Food Sci. Technol. Res..

[24] Clinical and Laboratory Standards Institute (2015). Methods for Antimicrobial Dilution and Disk Susceptibility Testing of Infrequently Isolated or Fastidious Bacteria.

[25] Bolger A.M., Lohse M., Usadel B. (2014). Trimmomatic: A flexible trimmer for Illumina sequence data. Bioinformatics.

[26] Bankevich A., Nurk S., Antipov D., Gurevich A.A., Dvorkin M., Kulikov A.S., Lesin V.M., Nikolenko S.I., Pham S., Prjibelski A.D. (2012). SPAdes: A new genome assembly algorithm and its applications to single-cell sequencing. J. Comput. Biol..

[27] Seemann T. (2014). Prokka: Rapid prokaryotic genome annotation. Bioinformatics.

[28] Moura A., Tourdjman M., Leclercq A., Hamelin E., Laurent E., Fredriksen N., Van Cauteren D., Bracq-Dieye H., Thouvenot P., Vales G. (2017). Real-Time Whole-genome sequencing for surveillance of *Listeria monocytogenes*, France. Emerg. Infect. Dis..

[29] Page A.J., Cummins C.A., Hunt M., Wong V.K., Reuter S., Holden M.T., Fookes M., Falush D., Keane J.A., Parkhill J. (2015). Roary: Rapid large-scale prokaryote pan genome analysis. Bioinformatics.

[30] Nguyen L.T., Schmidt H.A., von Haeseler A., Minh B.Q. (2015). IQ-TREE: A fast and effective stochastic algorithm for estimating maximum-likelihood phylogenies. Mol. Biol. Evol..

[31] Letunic I., Bork P. (2019). Interactive Tree of Life (iTOL) v4: Recent updates and new developments. Nucleic Acids Res..

[32] Olson R.D., Assaf R., Brettin T., Conrad N., Cucinell C., Davis J.J., Dempsey D.M., Dickerman A., Dietrich E.M., Kenyon R.W. (2023). Introducing the Bacterial and Viral Bioinformatics Resource Center (BV-BRC): A resource combining PATRIC, IRD and ViPR. Nucleic Acids Res..

[33] Camargo A.C., Moura A., Avillan J., Herman N., McFarland A.P., Sreevatsan S., Call D.R., Woodward J.J., Lecuit M., Nero L.A. (2019). Whole-genome sequencing reveals *Listeria monocytogenes* diversity and allows identification of long-term persistent strains in Brazil. Environ. Microbiol..

[34] Sullivan M.J., Petty N.K., Beatson S.A. (2011). Easyfig: A genome comparison visualizer. Bioinformatics.

[35] Van Stelten A., Simpson J.M., Ward T.J., Nightingale K.K. (2010). Revelation by single-nucleotide polymorphism genotyping that mutations leading to a premature stop codon in *inlA* are common among *Listeria monocytogenes* isolates from ready-to-eat foods but not human listeriosis cases. Appl. Environ. Microbiol..

[36] Lee S., Chen Y., Gorski L., Ward J., Osborne J., Kathariou S. (2018). *Listeria monocytogenes* source distribution analysis indicates regional heterogeneity and ecological niche preference among serotype 4b clones. MBio.

[37] Painset A., Björkman J.T., Kiil K., Guillier L., Mariet J.F., Félix B., Amar C., Rotariu O., Roussel S., Perez-Reche F. (2019). LiSEQ–whole-genome sequencing of a cross-sectional survey of *Listeria monocytogenes* in ready-to-eat foods and human clinical cases in Europe. Microb. Genom..

[38] Moura A., Criscuolo A., Pouseele H., Maury M., Leclercq A., Tarr C., Björkman J.T., Dallman T., Reimer A., Enouf V. (2016). Whole genome-based population biology and epidemiological surveillance of *Listeria monocytogenes*. Nat. Microbiol..

[39] Marcus R., Hurd S., Mank L., Mshar P., Phan Q., Jackson K., Watarida K., Salfinger Y., Kim S., Ishida M.L. (2009). Chicken salad as the source of a case of *Listeria monocytogenes* infection in Connecticut. J. Food Prot..

[40] Mughini-Gras L., APaganini J., Guo R., Coipan C.E., Friesema I.H.M., van Hoek A.H.A.M., van den Beld M., Kuiling S., Bergval I., Wullings B. (2025). Source attribution of *Listeria monocytogenes* in the Netherlands. Int. J. Food Microbiol..

[41] Filipello V., Mughini-Gras L., Gallina S., Vitale N., Mannelli A., Pontello M., Decastelli L., Allard M.W., Brown E.W., Lomonaco S. (2020). Attribution of *Listeria monocytogenes* human infections to food and animal sources in Northern Italy. Food Microbiol..

[42] Zhang P., Ji L., Wu X., Chen L., Yan W., Dong F. (2024). Prevalence, genotypic characteristics, and antibiotic resistance of *Listeria monocytogenes* from retail foods in Huzhou, China. J. Food Prot..

[43] Soge O.O., Beck N.K., White T.M., No D.B., Roberts M.C. (2008). A novel transposon, Tn*6009*, composed of a Tn*916* element linked with a *Staphylococcus aureus mer* operon. J. Antimicrob. Chemother..

[44] Gómez D., Azón E., Marco N., Carramiñana J.J., Rota C., Ariño A., Yangüela J. (2014). Antimicrobial resistance of *Listeria monocytogenes* and *Listeria innocua* from meat products and meat-processing environment. Food Microbiol..

[45] Wang Y., Ji Q., Li S., Liu M. (2021). Prevalence and genetic diversity of *Listeria monocytogenes* isolated from retail pork in Wuhan, China. Front. Microbiol..

[46] Anwar T.M., Pan H., Chai W., Ed-Dra A., Fang W., Li Y., Yue M. (2022). Genetic diversity, virulence factors, and antimicrobial resistance of *Listeria monocytogenes* from food, livestock, and clinical samples between 2002 and 2019 in China. Int. J. Food Microbiol..

[47] Liu X., Gao B., Li Z., Liang Y., Shi T., Dong Q., Chen M., Wu H., Zhang H. (2025). Research on the genetic polymorphism and function of *inlA* with premature stop codons in *Listeria monocytogenes*. Foods.

[48] Magagna G., Gori M., Russini V., De Angelis V., Spinelli E., Filipello V., Tranquillo V.M., De Marchis M.L., Bossù T., Fappani C. (2023). Evaluation of the virulence potential of *Listeria monocytogenes* through the characterization of the truncated forms of Internalin, A. Int. J. Mol. Sci..

[49] Kim H., Marquis H., Boor K.J. (2005). σ B contributes to *Listeria monocytogenes* invasion by controlling expression of *inlA* and *inlB*. Microbiology.

[50] Phelps C.C., Vadia S., Arnett E., Tan Y., Zhang X., Pathak-Sharma S., Gavrilin M.A., Seveau S. (2018). Relative roles of Listeriolysin O, InlA, and InlB in *Listeria monocytogenes* uptake by host cells. Infect. Immun..

